# Utility of a Leadless Pacemaker as a Backup to Left Ventricle–only Pacing in a Patient with Prior Device-related Severe Tricuspid Regurgitation

**DOI:** 10.19102/icrm.2019.100706

**Published:** 2019-07-15

**Authors:** Oscar Garza Ovalle, Jared Liebelt, Adrian Garza Ovalle, Amy Kaufman, Jay Alexander, Mark Metzl

**Affiliations:** ^1^Department of Cardiology, University of Chicago/NorthShore University Health System, Evanston, IL, USA; ^2^Department of Cardiology, NorthShore University HealthSystem, Evanston, IL, USA; ^3^Department of Medicine, Universidad de Montemorelos, Montemorelos, Nuevo Leon, Mexico; ^4^Department of Medicine, University of Chicago/NorthShore University Health System, Evanston, IL, USA

**Keywords:** Leadless, pacing, tricuspid

## Abstract

The contribution of endocardial cardiac device leads to severe tricuspid regurgitation (TR) has become increasingly recognized. Current strategies for treating cardiac device lead–related TR have limitations. We present a case of a pacemaker-dependent patient with severe TR as a complication of multiple cardiac device leads who underwent laser lead extraction, which was followed by implantation of a dual-chamber pacemaker with a coronary sinus lead for left ventricular pacing and a leadless transcatheter pacemaker for backup right ventricular (RV) pacing. This report represents one of the first cases of a leadless pacemaker implanted for RV backup pacing, highlighting the possibility of future biventricular pacing therapy (with a leadless pacemaker in VVT mode) without endocardial leads crossing the tricuspid valve.

## Introduction

Pocket- and lead-related complications are commonly encountered in cases of traditional transvenous pacemaker implantation.^1^ A leadless intracardiac transcatheter pacing system was designed to avoid these complications.^2^ In patients with severe tricuspid regurgitation, normal left ventricular ejection fraction, and pacemaker dependency in whom a right ventricular (RV) lead is contraindicated, single-site ventricular pacing via the coronary sinus (CS) has been proven to be effective and safe in a small retrospective study.^3^ We present a case of a pacemaker-dependent individual with severe tricuspid regurgitation (TR) as a complication of transvenous pacing leads who underwent lead extraction with eventual implantation of a CS lead for left ventricular pacing and a leadless pacemaker for backup RV pacing. To our knowledge, this is the first reported case of a leadless pacemaker implanted for RV backup pacing.

## Case presentation

**Figure 1:**
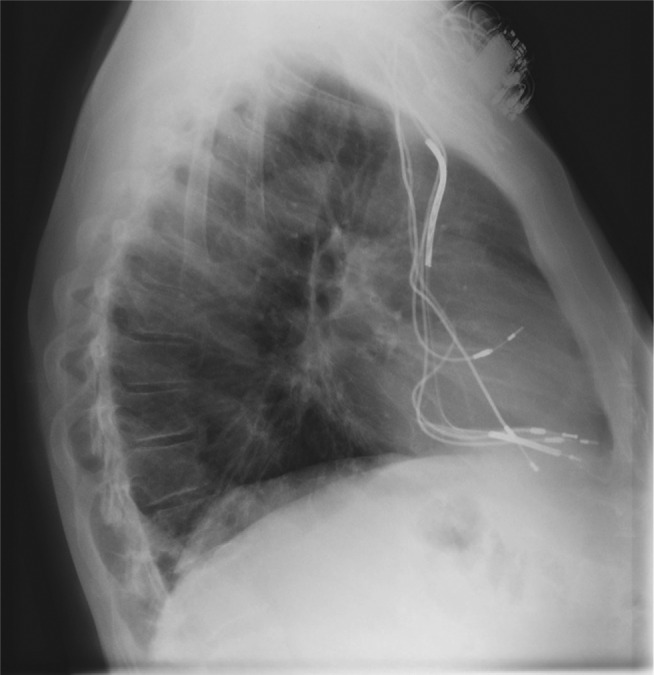
Initial lateral chest X-ray showing four leads crossing the tricuspid valve and causing severe TR.

An 86-year-old male with a medical history of complete atrioventricular block and atrial fibrillation had had a right-sided pacemaker implanted in 1983 with two RV leads. In 1997, he was upgraded to a left-sided dual-chamber pacemaker. He subsequently developed sustained monomorphic ventricular tachycardia (nonischemic) and his device was again upgraded to a dual-chamber defibrillator, similarly located on the left side. In 2001, he underwent successful ventricular tachycardia ablation. He presented last year to our hospital with a three-month history of worsening dyspnea on exertion (less than 50 feet), severe orthopnea, and lower-extremity edema. At this point, he was carrying two right-sided abandoned RV pacing leads, one left-sided right atrial lead, one left-sided RV pacing lead, and an RV defibrillator lead (ie, four leads crossing the tricuspid valve) **(Figure 1).**

Physical examination revealed a grade 3/6 holosytolic apical murmur, marked jugular venous distention, and grade 3+ pitting edema. Initial laboratory test results were remarkable for a pro–brain natriuretic peptide value of 786 pg/mL. Transesophageal echocardiography demonstrated a normal left ventricular ejection fraction and severe tricuspid regurgitation resulting from impingement by the pacing wires on the septal leaflet and poor leaflet coaptation. Right-sided heart failure secondary to severe TR was diagnosed. After the patient was aggressively diuresed, the decision was made to proceed with lead extraction with subsequent surgical tricuspid valve repair if no significant improvement was seen following lead removal. He underwent successful laser lead extraction of all four leads traversing the tricuspid valve. He decided against having another defibrillator implanted (ie, any implantable cardioverter-defibrillator including a subcutaneous one).

With the hope that his severe TR would improve without a pacemaker lead disrupting leaflet coaptation, pacemaker reimplantation was performed, with a bipolar lead placed distally in a CS branch and plugged into the RV port of a dual-chamber pacemaker (Adapta™ ADDR01; Medtronic, Minneapolis, MN, USA). The day following this procedure, however, the patient developed a rare but discomforting stimulation of the phrenic nerve from the CS lead at 25 beats to 40 beats per day. Alternate CS pacing vectors were tested, but the thresholds with these options were found to be not as good. CS lead output was decreased to improve the patient’s comfort, but this decrease raised the concern for a loss of ventricular capture in this pacemaker-dependent patient.

An array of next steps was considered, including the addition of a His-bundle lead, an epicardial lead, a leadless pacemaker, and/or a second bipolar or quadripolar CS lead and/or the performance of CS lead revision. Despite the concern for crosstalk between the two devices, we agreed that the best approach was the addition of a transcatheter leadless pacemaker for RV backup pacing. Via a right femoral approach, a leadless transcatheter pacemaker system (Micra™ Transcatheter Pacing System; Medtronic, MN, USA) was implanted in the RV and placed in VVI mode for backup. No crosstalk between the two devices was observed at implantation or over the next full year of follow-up.

**Figure 2:**
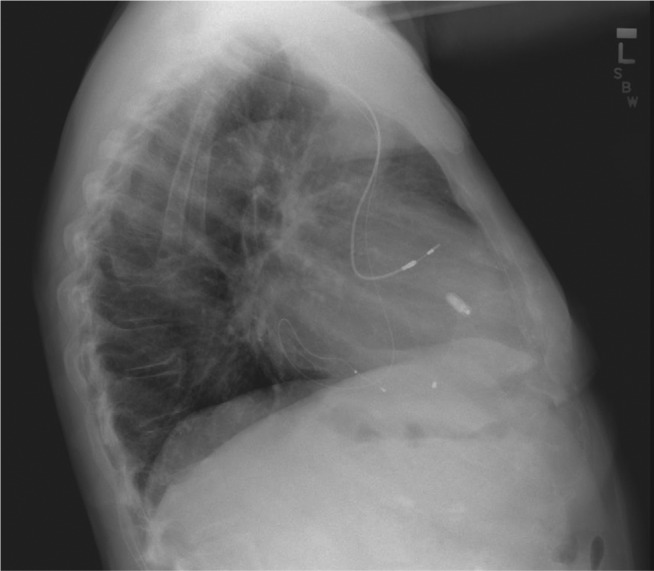
Lateral chest X-ray following initial lead extraction and leadless pacemaker insertion. Shown in the image is a right atrial lead, a CS lead, and a Micra™ pacemaker (Medtronic, Minneapolis, MN, USA).

Thereafter, the patient’s volume status significantly improved and he no longer required diuretics. His dyspnea on exertion abated. Repeat echocardiography at three months, six months, and nine months demonstrated moderate TR. No further tricuspid valve interventions were required **(Figure 2)**.

## Discussion

It is calculated that moderate or severe TR affects more than 1.6 million people in the United States alone. Gibson et al. first described the association between implanted cardiac devices and the increasing incidence of TR in 1980.^4^ In the present case, the patient had multiple pacemaker leads crossing the tricuspid valve that were prompting leaflet impingement and poor leaflet coaptation, with resulting severe TR. In the long-term, this caused right-sided heart failure and volume overload. Following lead extraction, his tricuspid valve disease ameliorated and he improved clinically.

The RV has traditionally been the preferred site of ventricular pacing, owing to the associated low risk of displacement, good reliability, and relative ease and safety of implantation.^5^ In patients with a history of tricuspid valve repair or replacement as well as in those with a history of pacemaker lead–related severe TR, operators who wish to avoid placing leads running across the tricuspid valve have several options. Single-site left ventricular pacing via the CS is a suitable option in this patient population, as it has been shown to be safe and reliable.^3^ Left ventricular leads may be placed surgically or percutaneously.^6^ His-bundle pacing has similarly emerged as a reasonable option to avoid traversing the tricuspid valve, as the lead in this procedure is most often placed on the atrial side of the tricuspid valve.^7^ Implantation of a leadless pacemaker was also entertained. While a leadless pacemaker that senses the atrium and paces the ventricle is under investigation at this time, current commercially available technology prevents us from preserving atrioventricular synchrony.^8^ Due to its widespread adoption and our experience with implanting pacing leads in the CS for cardiac resynchronization therapy, CS lead implantation was our first choice in the present case. Ultimately, the CS lead had a stable and excellent threshold; however, symptomatic phrenic nerve stimulation was observed when the pacing output was programmed with an adequate safety margin. After exploring other pacing vectors, we decreased the pacing output to improve the patient’s comfort, which, in turn, provoked another potential problem: the possible loss of ventricular capture.

At this point, we weighed the risks associated with reopening the pocket, accessing the venous system after a difficult extraction, and placing a His-bundle lead or a CS lead in another branch that may or may not have acceptable thresholds, all in a completely pacemaker-dependent patient.^9^ We ultimately decided against all of these options and instead opted to place a leadless pacemaker.

Leadless pacemakers were designed to avoid the pocket- and lead-related complications associated with traditional pacemakers.^2^ The Micra™ leadless transcatheter pacemaker system (Medtronic, Minneapolis, MN, USA) has demonstrated long-term safety and performance.^10^ Patients with this pacemaker were shown to demonstrate a 48% reduction in the risk of major complications as compared with control patients with transvenous systems, driven in part by a reduction in hospitalizations and system revisions.^10^ This reduced risk of complications was observed to an even greater extent (63%) in a real-world population due to an improved rate of pericardial effusion.^11^ The aforementioned leadless pacemaker was designed as a single-chamber pacemaker for patients with symptomatic sinus node dysfunction and a high degree of atrioventricular block as an alternative to dual-chamber pacing when lead placement is contraindicated or difficult to achieve. It is, however, contraindicated in patients with another functioning device, due to the potential for crosstalk that could lead to pacemaker inhibition.

In the case of our patient, whose options were limited, we opted for RV backup pacing using the Micra™ pacemaker (Medtronic, Minneapolis, MN, USA). Currently, there are no trials to support the use of this leadless pacemaker as an RV backup pacer; however, once implanted, both pacemakers appeared to be functioning adequately and they were thoroughly reviewed to avoid leadless pacing inhibition in the case of subthreshold CS lead pacing. The patient has demonstrated significant improvement to date.

## Conclusion

We believe this is one of the first reported cases of a leadless pacemaker implanted for RV backup pacing in a pacemaker-dependent patient with a CS lead also implanted for single-site pacing. The lack of device crosstalk further raises the possibility of this approach forming the basis of future biventricular pacing therapy (with a leadless pacemaker in VVT mode) without endocardial leads traversing the tricuspid valve.
